# Continuity of essential health services in the context of COVID-19: the Eastern and Southern Africa Regional continuity of essential services sub-working group

**DOI:** 10.11604/pamj.supp.2022.41.2.28000

**Published:** 2022-03-29

**Authors:** Trufosa Mochache, Maureen Momanyi, Ida-Marie Ameda, Fatima Gohar, Michael Ebele, Fanuel Odhiambo, Tasiana Mzozo, Hana Bekele, Mwangi Waituru, Miriam Nanyunja

**Affiliations:** 1 WHO Emergency Hub for East and Southern Africa, Nairobi, Kenya,; 2 UNICEF, East and Southern Africa Regional Office, Emergency Hub for East and Southern Africa, Nairobi, Kenya,; 3 UNFPA Eastern Sub Regional Office, Nairobi, Kenya,; 4 WHO Regional Office for Africa, Cité du Djoué, Brazzaville, Republic of Congo,; 5 VSO, International, Nairobi, Kenya

**Keywords:** Continuity of Essential Health Services, COVID-19, East and Southern Africa

## Abstract

COVID-19 cases have continued to increase globally putting intense pressure on health systems, including in the East and Southern African (ESA) region, which bears the brunt of the continent´s cases, and where many health systems are already weak or overstretched. Evidence from the West Africa Ebola disease outbreak and early estimates for COVID-19 show that indirect impacts due to disruptions in access to essential health services can result in even higher mortality than that directly related to the outbreak. In March 2020, World Health Organisation (WHO) established a coordination mechanism to support ESA countries to enhance their response to COVID-19. Technical working groups were established, including a subgroup addressing continuity of essential health services. In this article, the development, activities and achievements of the subgroup over the past six months are reviewed and presented as a model for collaborative action for optimal service delivery in the context of COVID-19 and potentially, during other infectious disease outbreak responses.

## Essay

### Background

Within the World Health Organization Regional Office for Africa (WHO-AFRO) region, increasing COVID-19 cases have put intense pressure on health systems. The ESA sub-region, where many health systems are already weak or overstretched, now bears the brunt of COVID-19 cases on the continent [1]. Besides direct outbreak related mortality, overwhelmed health systems may result in increases in indirect mortality as a result of reduced access to essential health services. This was the case in the West Africa Ebola Virus Disease (EVD) outbreak (2014-2015), where the number of deaths caused by disrupted access to essential maternal and newborn care, measles, malaria, HIV/AIDS, and tuberculosis exceeded direct EVD mortality [2, 3]. The 2020 Goalkeepers Report [4] highlights some of the negative effects of the COVID-19 pandemic on access to health care and key essential services such as vaccination, maternal health services, neonatal services, malaria, HIV, TB, sexual and reproductive health services. Access to these services shows significant decline during the COVID-19 pandemic with gains made over the last decades towards decreasing the rates of under-five and neonatal mortality, acute malnutrition and stunting, having plateaued, and may continue to regress if action is not taken [4, 5]. The pandemic has highlighted the need for countries to balance the demands of outbreak response, while ensuring continued delivery of essential health and nutrition services, to mitigate the risk of system collapse. In March 2020, WHO conducted a regional partners´ meeting establishing a coordination mechanism to support ESA countries to enhance their response to COVID-19. Technical working groups (TWGs) were established, including a subgroup specifically addressing continuity of essential health services. In this article, the development, activities and achievements of this subgroup over the past six months (March to September 2020) are reviewed and presented as a model for collaborative action for optimal service delivery in the context of COVID-19 and potentially, during other infectious disease outbreak responses.

### The continuity of essential services sub-working group: mandate, membership and approach

The Continuity of Essential Services Sub-Working Group (CES-SWG) was established in April 2020 to provide an innovative, inter-agency platform to support ESA countries to sustain the delivery of essential health and nutrition services in the context of the COVID-19 pandemic. It brings together a range of regional stakeholders to provide collective strategic technical support to countries to mitigate the collateral impact of COVID-19 on continuity of essential health and nutrition services. It is one of four sub-working groups under the sub-regional Case Management TWG, established to provide dedicated support to ESA countries to respond to COVID-19 and its indirect health impacts.

Membership of the sub-working group was initially drawn from WHO, UNICEF and UNFPA but has since expanded to include Africa CDC, other United Nations (UN) agencies including UN Refugee Agency (UNHCR), International Organization for Migration (IOM), World Food Programme (WFP), UN Office for the Coordination of Humanitarian Affairs (UNOCHA); implementing partners working in member states including Voluntary Service Overseas (VSO) International, save the children, action against hunger and European Commission´s Humanitarian Aid department (ECHO) representing regional donors. Given the group´s technical focus, individuals with specific expertise and responsibilities for the health and nutrition service delivery were nominated to represent their institutions.

The Services Sub-Working Group (SWG) holds weekly meetings using web-based conferencing platforms, during which updates and emerging issues are discussed, which are presented to the broader Sub-regional Case Management Technical Working Group that in turn elevates concerns to the Health Partner´s Group Meeting for joint action including policy advocacy. The SWG is supported by a core team from WHO, UNFPA and UNICEF, with coordination support from Office for the Coordination of Humanitarian Affairs (OCHA).

The group allows for a vibrant discourse tackling issues pertaining to continuity of essential services informed by the group members´ knowledge and interaction with country level counterparts. It allows for the interrogation of existing global, regional and national health and nutrition approaches to service delivery, exploring their adaptation to the COVID-19 context in the subregion. The group engages with member states in a coordinated manner to review progress, guide on new approaches, table emerging issues and resolve system bottlenecks to ensure sustained delivery of quality health services.

### Approach to technical support to countries

The sub-working group´s activities are built around three main themes: 1) *Advocacy for continuation of essential health and nutrition services*namely: reproductive health (including gender-based violence); maternal, newborn, child and adolescent health services; nutrition; management of communicable & non-communicable diseases; management of trauma and other emergencies; and services for migrant and other vulnerable populations. 2) *Timely monitoring of disruptions in continuity of essential services due to the COVID-19 pandemic and response interventions and their impact on health and nutrition services*. 3) Supporting countries to recognize and address causes of disruptions to restore service delivery and minimize indirect mortality. The CES-SWG agenda helps in ensuring countries remain on course to achieving global, regional and national health-related targets including, but not limited to the Sustainable Development Goals (SDGs), even in the context of COVID-19.

### Accomplishment

**Figure 1:**
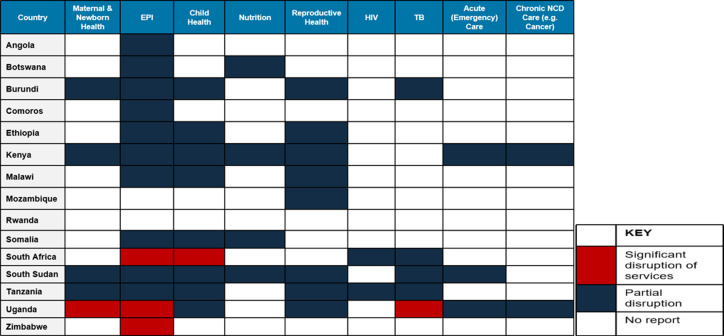
summary analysis on the qualitative reports on disruption of essential services: outcome of the Eastern and Southern Africa Regional continuity of essential services sub-working group review

*Monitoring disruptions in continuity of essential health and nutrition services:*as a first step the SWG established a mechanism for monitoring disruptions that could be due to the COVID-19 pandemic and response interventions using both qualitative and quantitative approaches. The qualitative method monitors disruptions through assessment of qualitative reports from media sources and partner reports, using the event-based surveillance approach, while quantitative data is obtained through systematic (primarily programmatic) data collection processes and national health management information systems. A matrix was developed to serve as a repository of all reports including from partner field reports, mainstream media and published articles all of which were presented during the weekly meetings, triangulated with partner field experiences and validated. This was invaluable at a time when systematic collection and visualization of essential service delivery at regional level were still in development. It provided insights on which countries were experiencing disruption in essential health service delivery and the causes of the disruptions, informing action from the group including country follow up for technical assistance, advocacy and linkages with other regional working groups. Early findings from the qualitative reports revealed disruptions, with the most affected services being immunization and other child and maternal health services across majority of the countries in the ESA region (Figure 1).

**Figure 2:**
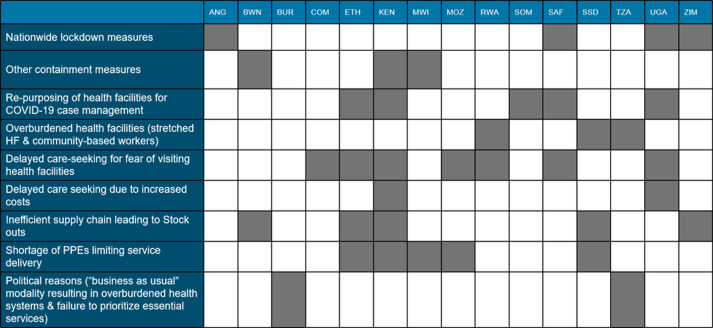
main causes of disruptions in continuity of essential health and nutrition services during the COVID-19 pandemic: outcome of the Eastern and Southern Africa Regional continuity of essential services sub-working group review

Although in most cases the disruptions were noted to be partial and restricted to urban areas, there were instances where these were nationwide owing to lockdown measures. On the one hand supply factors played a significant role in impeding service delivery in several countries; including factors such as re-purposing of health facilities for COVID-19 case management; over-burdened health facilities with stretched facilities and health workers and inefficient supply chain mechanisms leading to shortages in essential supplies including PPEs (Figure 2). Demand-side factors such as delayed care-seeking out of fear of visiting health facilities for fear of contracting COVID-19, stigma-related issues, myths around vaccination and increased costs of transportation, contributing to low uptake of services (Figure 2).

Beyond the health system supply and demand bottlenecks, the qualitative reports also revealed compounding factors that were either pre-existing or emerging that were contributing to disruption of essential health services. These included the floods across the greater horn of Africa resulting in displaced vulnerable population groups and predisposing communities to climate sensitive and water-borne diseases yet disrupting access to health facilities, conflict in some countries creating complex emergencies, outbreaks of epidemic prone diseases such as cholera, yellow fever and haemorrhagic fevers; food insecurity and drought as well as the economic downturn. Discussions stemming from these qualitative reports´ were used to inform coordinated CES-SWG actions including innovative contextualized country support actions. In addition, the analysis inspired programmatic systematic monitoring and analysis of the status of continuity of essential services in the region.

*Interim CES Guidance:* the CES-SWG´s next step was the development of regional guidance which are contextualized and built on global interim guidance for essential health services, aimed at helping countries address challenges in ensuring sustained essential health and nutrition services [6-9]. The East and Southern Africa Region Joint Interim Guidance on Continuity of Essential Health and Nutrition Services during the COVID-19 Pandemic [10] guides countries on: 1) identifying context relevant essential services; 2) optimizing service delivery settings and platforms; 3) addressing health workforce capacity and needs, including safety, in the context of the COVID-19 response, while ensuring continuity of essential services; 4) promoting ways to ensure uninterrupted supply and distribution of essential health and nutrition commodities; 5) monitoring continuity of essential services. The guidance document was launched through a global webinar on 12 June 2020, with over 300 participants from 41 African member states and has been adapted by several countries since then.

*CES pulse survey:*to facilitate tailored country support following the dissemination of the joint guidance on continuity of essential services during COVID-19 pandemic, a quick pulse survey was conducted with 22 countries in Eastern and Southern Africa. The four-question survey sought to find out about: 1) presence of a pillar/sub-pillar on CES within the national COVID-19 taskforce; 2) presence of an overarching policy or guidelines on continuity of essential services during COVID 19 pandemic; 3) existence of a mechanism to monitor continuity of essential services; 4) availability of essential commodities for ensuring continuity of essential services including personal protective equipments (PPEs).

The survey yielded a 91% response rate (20/22) with most of the countries (64%) reporting to have a CES pillar/sub-pillar in place. More than half of the countries (59%) had adapted or developed national guidance documents on continuity of essential services, while four countries were in the process of adapting/developing guidance. Almost all the countries had instituted mechanisms for monitoring CES, and nine (9) countries reported having disruptions in the delivery of sexual and reproductive health, child health, immunization, malaria, TB and HIV services. Over 80% of the countries (18/22) reported shortage of PPE or other commodities such as essential medicines. The CES-SWG used the findings of the rapid survey to prioritize countries for technical assistance including advocacy to address system bottlenecks, development of relevant policy or guidance on CES and strengthening capacities of in-country teams.

*Logistics to ensure continuity of essential services in countries:* the SWG leveraged linkages with the sub-regional logistics Thematic Working Group (TWG) to advocate for country needs and conduct an in-depth analysis on country-specific supply chains for essential commodities including a commodity gap analysis.

**Cross country learning through deep dives:** the Regional Case Management Working Group, invited case management teams from several countries to share their experiences in cross country deep dive sessions, focusing on the country profile, epidemiological situation, key actions on country preparedness and response covering all pillars including continuity of essential services; challenges; best practices and requests for support. The findings from this country engagement process guided regional partners on the support needed by specific countries in the COVID-19 response.

From the deep dives, it was evident that countries were struggling to sustain essential service delivery on one hand while accelerating COVID-19 preparedness and response actions on the other. For some countries, the pressures of the COVID-19 pandemic only exacerbated pre-existing health system challenges. The increasing risk of health care worker infections due to inadequate PPE and other factors was noted by all countries, with a concern that it would compound existing human resource for health challenges further impeding delivery of essential health services. Shortage of essential medicines was another recurring concern articulated by most countries, attributed to delayed procurement and delivery processes, operational obstacles due to lockdown and/or containment measures hindering importation as well as in country logistics challenges, such as inadequate forecasting and inequitable distribution. From the country deep dives, the Case Management TWG, of which the CES-SWG is a part, was able to identify and prioritize areas of support to countries in the sub-region in order to achieve an effective balance between the COVID-19 response and continuity of essential health and nutrition services.

### Challenges

The CES-SWG has faced a few challenges in execution of its work, however, these have not affected the operations of the group significantly. Group cohesion and good working relationships between the member institutions and countries within ESA had to be developed quickly to enable effective performance of the group´s work. This required time and dedication of the members amidst their other work at their institutions, however, with effective leadership and commitment to the noble cause, this was achieved. Due to conflicting demands within the member institutions as well as from national counter parts, at times deliverables including country engagement for support were delayed. Finally, the CES-SWG does not have an independent funding source or financing mechanisms, and is thus reliant on member institutions to undertake and agree upon actions in their own capacity. This limits the nature and timeliness of support that can be given to countries.

### Discussion

The CES-SWG brought together technical officers, with different expertise and experiences together around a common goal of supporting countries to ensure continuity of essential health and nutrition services in order to minimize on indirect mortality during COVID-19 pandemic. The strength of a multi-disciplinary, multi-agency team, such as that in the CES-SWG, is its inherent capacity to comprehensively interrogate and address cross-sectoral issues impacting, in this case, continuity of essential services in the context of the COVID-19 pandemic in member countries using different channels available to the member institutions. The team was able to identify key obstacles to continuity of essential health services and through the different networks, country offices, and communication channels available to the different agencies advocate for and support address of these obstacles. There were thus substantial gains in getting the different agencies to work together. Keeping such multi-agency, multi-disciplinary team working together requires good leadership. The leadership provided jointly by WHO and UNFPA encouraged active participation of all and enabled development of group cohesion. As the pandemic continues, it is critical that this is maintained and that the group continues to support emerging issues affecting the continuity of health services.

The innovative approach to monitoring disruptions of essential health services using both qualitative and quantitative approaches enabled quick identification of disruptions and their causes; countries were engaged and supported to address most of these immediately. The qualitative approach was built on the principles of event-based surveillance as highlighted in the Third edition of the Integrated Disease Surveillance and Response Guideline [11] and the Africa Centers for Disease Control and Prevention (CDC) Framework for Event based surveillance [12]. Efforts by the regional surveillance technical working are ongoing to encourage and support countries to include monitoring disruptions of health services as a part of event-based surveillance. The quantitative data from the country health information management systems (HMIS), that was analysed monthly showed the quantitative changes in access and utilization of services compared to previous years.

In order to ensure continuity of essential health services, this has to be given as high a priority as is being given to the COVID-19 response, hence the importance of a pillar on continuity of essential services. In some countries however, it was noted that continuity of essential services was a sub-pillar under the case management pillar and the guidelines for this were integrated in the case management guidelines. As long as continuity of essential services is given high priority, we considered this adequate. It has been noted that even in countries with established pillars and guidelines on continuity of essential services, disruptions still occur due to some factors including health worker infections and need for health facility decontamination among others. Continuous qualitative monitoring is thus important for detection of such disruptions so that measures are taken to address them immediately and restore service delivery.

During public health emergencies, the area of medical logistics is often challenged, as has been reported by countries for COVID-19. The challenges in availability of medical commodities especially personal protective equipment, COVID-19 laboratory tests, and essential medicines were mainly due to global supply issues, inadequate forecasting, inadequate funding resulting in delayed placement of orders, and delayed delivery due to transportation challenges due to airport closures and restrictions. There is need to strengthen the medical logistics capacity of countries and partners to enable more effective logistical support to COVID-19 and other public health emergencies. The regional logistics technical working group, in collaboration with the other technical working groups, is making tremendous efforts in this regard.

### Conclusion

Since its formation in March 2020, the CES-SWG has established itself as a unique forum for collaboration of partners working in the region in health, nutrition, child protection and humanitarian programmes to discuss regionally relevant issues and innovative approaches to supporting member countries in sustaining essential health and nutrition services in the context of the COVID-19 pandemic, and may form a model for partnership and coordination in ensuring continuity of essential health services. The substantial gains made by the group in this relatively short period of time are due to the groups´ cohesion around a common goal, good working relationships built within the group, investments and commitment made by members and accommodative leadership. The ability of group members to proactively engage with country teams continues to drive progress in prompt, coordinated, country level engagement and action, as it allows for rapid validation of issues and contextual discussions on innovative approaches. The diverse membership of the group and linkages to other sub-regional technical working groups further enables broader advocacy on topical issues pertaining to continuity of essential services and support of country needs. The structure and approach of the CES-SWG has provided a model for regional response tackling of what would have otherwise remained a peripheral issue, especially given the competing pressures of responding to a global pandemic. Consideration should be given to sustaining and further developing this group to support focus on continuity of essential health services during other emergencies in the ESA region.
